# Kimura Disease Manifesting as Synchronous Bilateral Parotid Swelling in a Young Middle-Eastern Patient

**DOI:** 10.1155/2014/648607

**Published:** 2014-11-24

**Authors:** Fatemah Faras, Fawaz Abo-Alhassan, Khalid Al-Sebeih, Jassem Bastaki

**Affiliations:** ^1^Department of ENT, Sabah and Zain Hospital, Ministry of Health, 40188 Mishref, Kuwait; ^2^Department of Surgery, Al-Adan Hospital, Ministry of Health, 40188 Mishref, Kuwait; ^3^Department of Pathology, Sabah and Kuwait Cancer Control Center, Ministry of Health, 40153 Mishref, Kuwait

## Abstract

Kimura disease is a rare, benign, chronic inflammatory swelling of the subcutaneous tissue, lymph nodes, and glandular tissue. Characteristic features of the disease include, but not limited to, painless subcutaneous head and neck swelling, blood and tissue eosinophilia, and markedly elevated immunoglobulin E (IgE) levels. Herein, we report a rare case of Kimura disease manifesting as synchronous bilateral parotid swelling of 12 years duration in a 33-year-old Middle-Eastern man. To our knowledge only few cases have been reported in the literature involving bilateral parotid glands, and this is the first case to be reported in the Middle East.

## 1. Introduction

Kimura disease (KD) is a rare condition that was initially described in 1937 in China by Kim and Szeto [1]. In 1948 Kimura et al. reported similar cases in Japan and further elaborated on its histopathologic features [2]. KD is a chronic inflammatory disease of an etiology that is not entirely understood. The disease usually manifests with unilateral swelling in the soft tissues of the head and neck, including salivary glands and lymph nodes, and is associated with peripheral blood eosinophilia. The lesions have no malignant potential [3, 4]. We report this rare case of Kimura disease in a young Middle-Eastern man who presented with synchronous involvement of bilateral parotid glands with a serum analysis that revealed elevated eosinophil count and IgE level. The clinical presentation, histopathologic features, differential diagnosis, and therapy are discussed in this paper.

## 2. Case Presentation

A 33-year-old Kuwaiti male presented to our clinic with swelling in the left parotid region for the past 12 years. The swelling rapidly increased in size in the last few months reaching 2 × 2 cm. There was no history of fever, pain, pruritis, unexplained weight loss, or renal symptoms.

**Figure 1:**
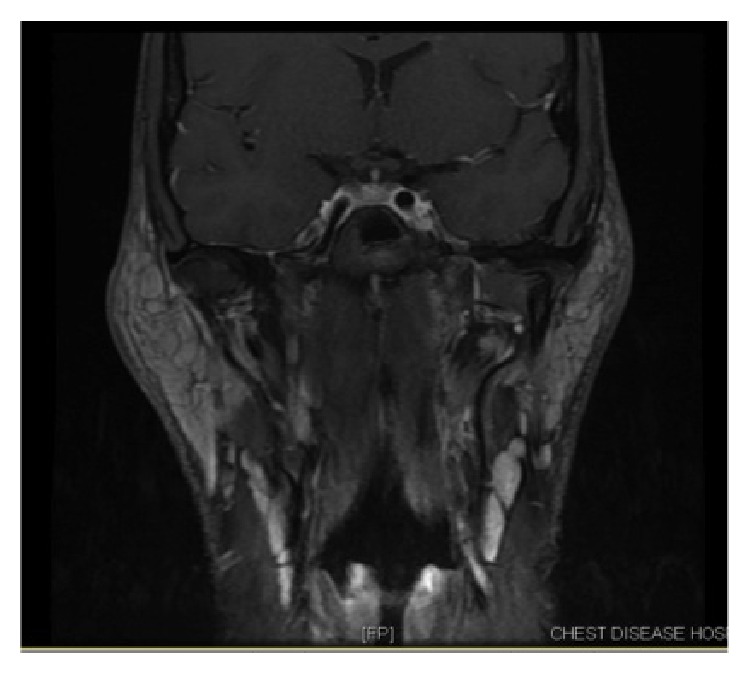
Preoperative MRI T1-weighted coronal cut: bilateral enlarged parotids and intraglandular lymph nodes.

**Figure 2:**
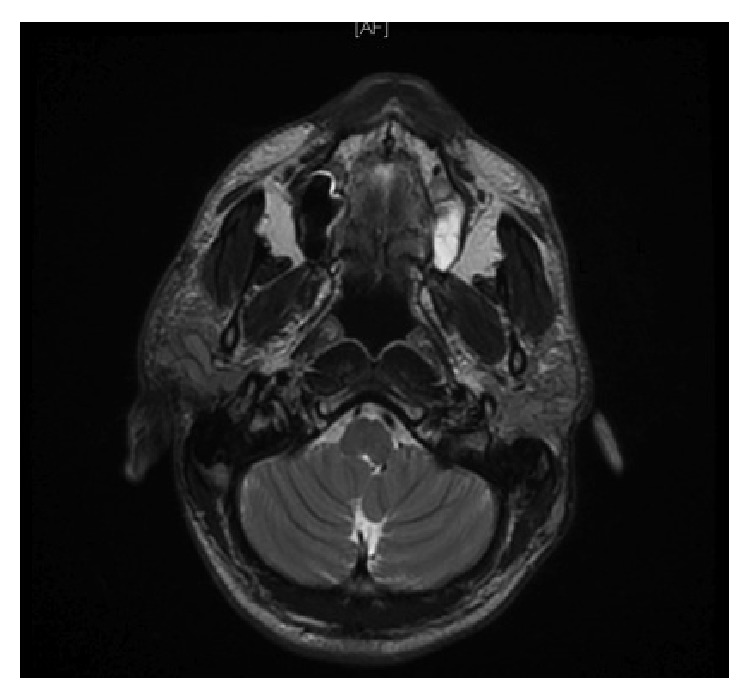
Preoperative MRI T2-weighted axial cut: bilateral hyperintense enlarged parotids, with thickened overlying subcutaneous tissue.

In the past history, the patient reported to have undergone a right parotidectomy 7 years back for a similar mass on the contralateral side. Unfortunately, the only available records for us to review were the initial biopsy report and the preoperative magnetic resonance imaging (MRI). The MRI, done preoperatively, showed bilateral enlarged parotid glands, with multiple enlarged intraparotid lymph nodes. The findings were more prominent on the right side (Figures 1 and 2). The fine needle aspiration cytology (FNAC) and the incisional biopsy of the right parotid tissue showed extensive fibrosis with focal inflammatory cell infiltrates composed of lymphoid cells and numerous eosinophils, suggesting the diagnosis of KD. The patient was previously treated with steroid therapy with remission and relapse after steroid cessation.

During the current admission, a comprehensive head and neck examination revealed a soft, nontender, mobile 2 × 2 cm left parotid swelling. No lymphadenopathy or facial nerve palsy was noted. The head and neck exam was otherwise insignificant.

Laboratory testing showed normal white blood cell count with eosinophilia (7%). The renal function test along with the urine routine was normal. IgE level was highly elevated to 625.7 IU/mL (normal range 11–162 IU/mL).

**Figure 3:**
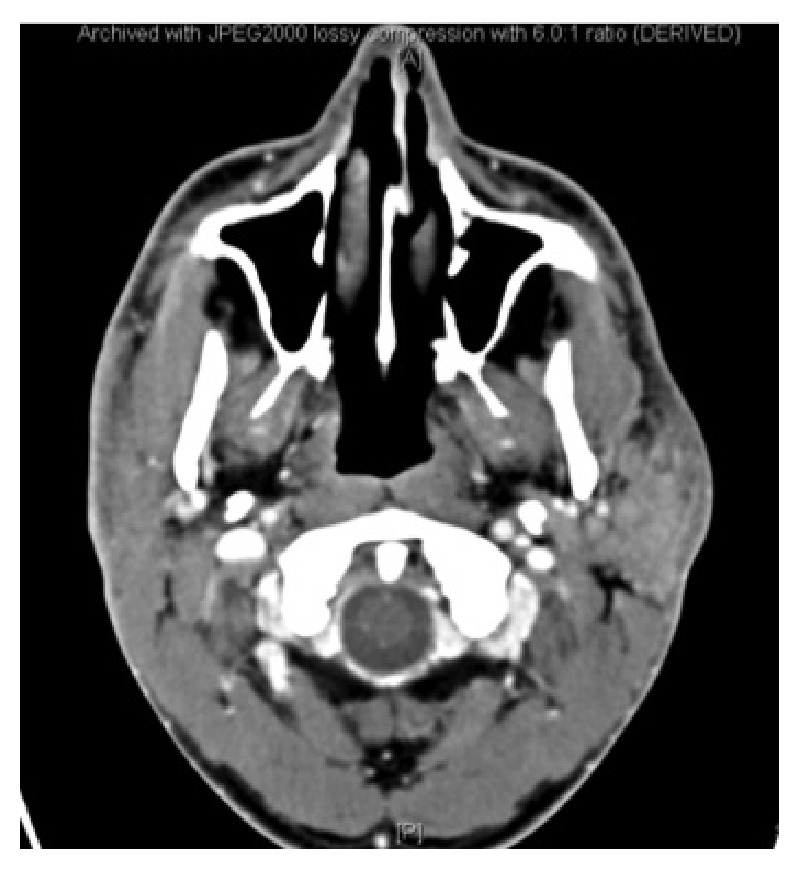
CT axial cut: left parotid gland enlargement with heterogeneous density, multiple different sized intraglandular lymph nodes.

Radiological imaging, upon admission, is illustrated in Figure 3. The computed tomography (CT) showed left parotid gland enlargement with heterogeneous density, multiple different sized intraglandular lymph nodes.

The patient underwent an elective left superficial parotidectomy through a modified Blair's incision. Retrograde dissection of the parotid gland was done with preservation of the facial nerve. The postoperative period was uneventful and the facial nerve was intact.

**Figure 4:**
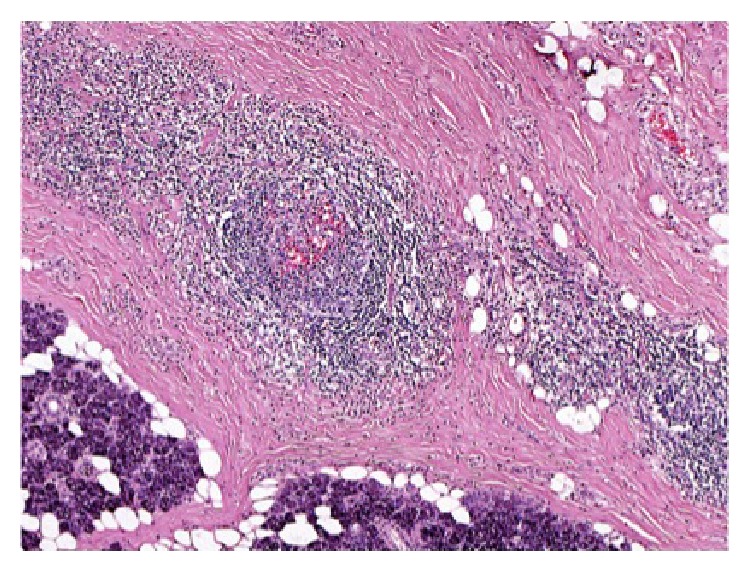
A diffuse chronic inflammatory cell infiltrate with germinal center formation and fibrosis (H&E staining, 40x).

**Figure 5:**
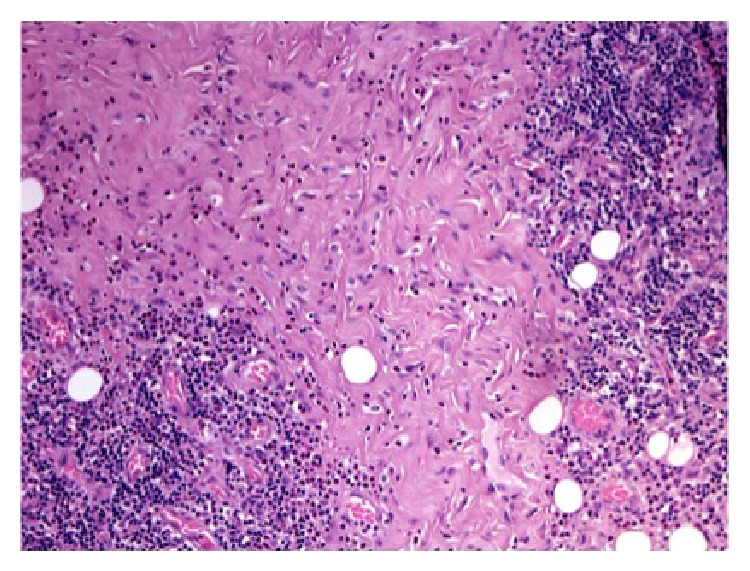
Scattered eosinophils are seen throughout (H&E staining, 100x).

**Figure 6:**
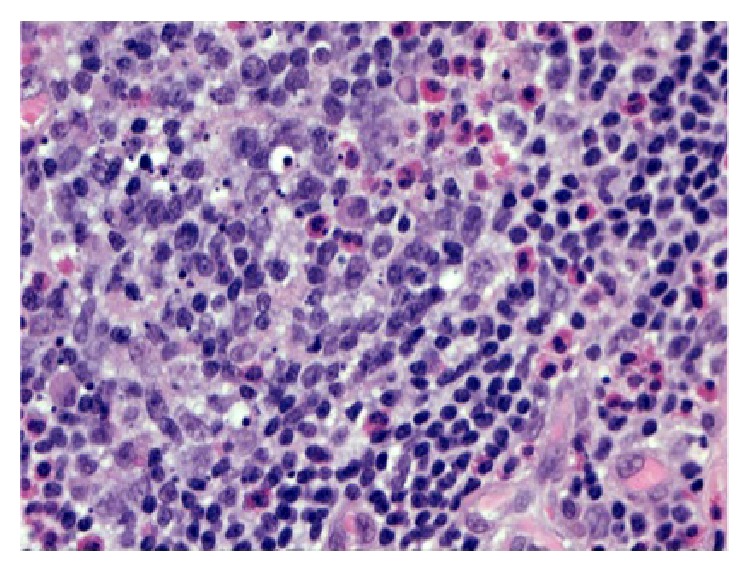
Eosinophils within germinal centers (H&E staining, 400x).

**Figure 7:**
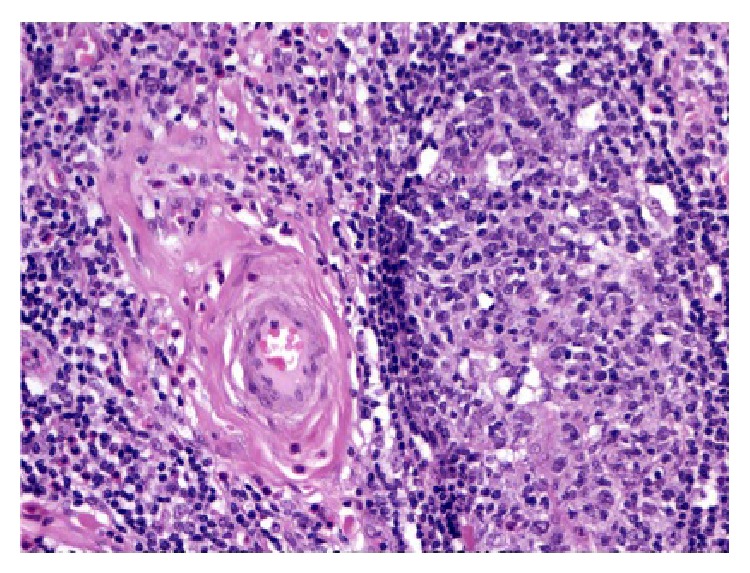
Perivascular fibrosis is seen involving scattered vessels (H&E, 200x).

The sections of the formalin fixed and paraffin embedded left superficial parotidectomy specimen showed extensive fibrosis of the parotid with loss of the normal parenchyma and a diffuse chronic inflammatory cell infiltrate (Figure 4). The infiltrate is composed of lymphocytes and plasma cells with prominent germinal center formation and eosinophilia (Figure 5). The process also extends outside the parotid superficially in a mass-like fashion. Interestingly, eosinophils were seen within the germinal centers (Figure 6) with rare eosinophilic abscesses. Mild fibrosis was also present around scattered vessels (Figure 7).

## 3. Discussion

Kimura's disease (KD) is a rare idiopathic chronic inflammatory disorder, affecting primarily Chinese and Japanese descents [1, 2]. Only a few cases have been reported from the West. This case is unique because, to the best of our knowledge, this is the first case of Kimura disease involving bilateral parotid glands in a Middle-Eastern male. KD most commonly occurs in young male adults in their second and third decades of life [5–7]. The head and neck region is the most common site for the disease manifestation (70%) mainly involving the subcutaneous tissue, parotid glands, and lymph nodes [8, 9]. Less often affected sites are the groin (15%), extremities (12%), and trunk (3%) [10].

The etiology of KD remains unclear though several theories have been proposed. It has been speculated that it could be a self-limited allergic or autoimmune reaction initiated by an unknown stimulus. It has also been proposed that viral infections or toxins may induce IgE mediated type 1 hypersensitivity resulting in the release of lymphokines [4, 11]. These theories are supported by the consistent laboratory findings of elevated blood eosinophils, and IgE levels, and the typical histologic picture of lymphoplasmacytic and eosinophilic infiltration. However no specific allergens have been identified [12].

The diagnosis of KD can be difficult because clinicians and pathologists are relatively unfamiliar with this rare disease, especially in Western countries. Typically, patients present with a long history of gradual increase in the size of a mass, in the head and neck region. The lesion usually is firm, painless or pruritic, and often involves the subcutaneous tissue, lymph nodes, or the salivary glands [13, 14]. However some cases have been reported to involve other parts of the body such as the oral cavity, conjunctiva, eyelid, tympanic membrane, skeletal muscle, prostate, and kidney [13].

Elevated peripheral blood eosinophils and IgE levels are quite characteristic laboratory findings of KD, but the exact diagnosis can only be revealed by a histologic examination of the tissue biopsy [13, 14]. Histologic examination characteristically shows dense fibrosis, capillary proliferation, lymphoid infiltration with reactive follicles, and pronounced eosinophilic infiltration. In some cases eosinophilic microabscesses can be seen [10]. Polykaryotic giant cells are commonly found. When fine needle aspiration cytology is used to diagnose KD, Hodgkin's disease can be suspected due to the polymorphous infiltrate with eosinophilia and presence of giant cells. However, the absence of Reed Sternberg cells helps rule it out [13].

It has been reported that KD is associated with renal disease more than the normal population [15]. Nephrotic syndrome is the most common and most significant systemic manifestation of KD [3]. In such cases, renal lesions have shown variety of histologic presentations such as minimal change disease, mesangioproliferative glomerulonephritis, focal segmental glomerulosclerosis, membranous nephropathy, and IgM and IgA nephropathy [14]. Our patient had neither renal involvement nor proteinuria.

Clinically, the differential diagnosis includes angiolymphoid hyperplasia with eosinophilia (ALHE), Kaposi's sarcoma, Sjögren syndrome with parotid involvement, Hodgkin's disease, tuberculosis, nodal metastasis, Warthin's tumor, and low grade angiosarcoma [3, 4, 10].

Some theories proposed that KD and ALHE are the same or different stages of the same disease process [3]. Similar features between the two diseases are male predominance, predilection for head and neck region, relative long course, and good prognosis. However, ALHE is different clinically in that the lesions are multiple dermal papular or nodular eruptions in older patients. Peripheral blood of ALHE is less frequently accompanied by eosinophilia. Histologically both have a proliferative vascular nature with eosinophilic and lymphoid infiltrates [3, 13, 16].

Treatment of KD is still controversial. Observation, steroid, radiotherapy, cryotherapy, cytotoxic therapy, and surgery have all been tried with different success rates [3, 17, 18]. Steroid therapy has shown relapses after withdrawal from treatment, and some patients have become refractory to it [19]. Radiotherapy has been tried but the usual benign course of the disease has discouraged its use [4]. However in cases that are steroid resistant or in young patients, radiotherapy has been used to prevent relapse and reduce the long-term side effects of steroid therapy [20, 21]. Surgical excision remains the treatment of choice even though recurrence is common [13, 20, 22, 23].
